# Drug-eluting stents versus bare-metal stents in the treatment of drug-refractory intracranial atherosclerotic disease: a retrospective single-center comparison

**DOI:** 10.1186/s42155-026-00654-2

**Published:** 2026-01-26

**Authors:** Philipp von Gottberg, Hans Henkes, Kamran Hajiyev, Michael Forsting, Andrei Filioglo, Hansjörg Bäzner, Ali Khanafer

**Affiliations:** 1 Neuroradiologische Klinik, Klinikum Stuttgart, Stuttgart, Germany; 2 Institut Für Diagnostische Und Interventionelle Radiologie Und Neuroradiologie, Universitätsklinikum Essen, Universität Duisburg-Essen, Essen, Germany; 3 Department of Neurosurgery, Institute of Emergency Care, Chisinau, Republic of Moldova; 4 Neurologische Klinik, Klinikum Stuttgart, Stuttgart, Germany

**Keywords:** Intracranial atherosclerotic disease, Stroke, Intracranial stenting, Drug-eluting stent, Neurovascular intervention

## Abstract

**Introduction:**

Due to several issues with endovascular treatment of intracranial symptomatic atherosclerotic disease (ICAD), international guidelines recommend drug therapy as first-line treatment since almost 10 years. Regardless of this, endovascular ICAD treatment has meanwhile progressed, significantly reducing periprocedural complication rates.

However, early in-stent restenosis (ISRS) remains, so far, unchallenged.

Cardiologists had been at a similar point in the evolution of cardiac artery stenting and challenged ISRS through drug-eluting stents (DES).

Therefore, 90d results and restenosis rates of patients treated with DES versus bare-metal stents (BMS) were compared to determine whether DES could also solve the neurovascular problem.

**Methods:**

All endovascular ICAD treatments in 2014–2022 at a single institution through DES were retrospectively compared to all BMS-treatments in regards to periprocedural complications and ISR rates. Perioperative procedures and drug regimen were comparable for all patients, follow-up was carried out 90 days after treatment through digital subtraction angiography, clinical status was recorded by a board certified neurologist.

**Results:**

Fifty-two patients were treated for ICAD with DES, 26 patients received ICAD-treatment with BMS. Patients’ demographics and comorbidities were comparable. The periprocedural and ISR-rates were significantly lower in DES compared to BMS.

**Discussion:**

The lower periprocedural complication rate may be due to less complexity of the DES system, while lower ISR in the DES group may be connected to reduced endothelium irritation and proliferation through the eluted sirolimus.

**Conclusion:**

In the population studied, DES achieved equal to better results in comparison to BMS. This study supports a re-evaluation of the role of endovascular ICAD-treatment as first line therapy in the light of the most recent developments on knowledge and techniques.

## Introduction

International guidelines recommend drug therapy as first-line treatment for intracranial symptomatic atherosclerotic diseases (ICAD) since almost 10 years, leading to a subsequent significant slowdown in scientific development and a decline in the corresponding treatment figures. It is only recently that—contrary to these recommendations based on study data from the early 2010s—endovascular treatments for ICAD have increased [1, 2] and scientific progress continues to be made [3–5]—particularly thanks to research groups in Asia [6–12].

A reason for this is that the high periprocedural morbidity and mortality of the milestone studies SAMMPRIS [13] and VISSIT [14] could be significantly reduced in follow-up studies through better patient selection, improved and new techniques and materials, encouraging neurointerventionalists to perform intracranial stenting.

However, the problem of the comparably high number of in-stent restenosis (ISRS) in SAMMPRIS and VISSIT has not yet been investigated or even solved to the same extent.

Neointimal hyperplasia, the proliferation of the vessel wall around the stent struts, is considered to be a main reason for ISRS [15]. In cardiology, the problem of ISRS of coronary stents has been challenged successfully already years ago by the introduction of drug-eluting stents (DES) and drug-coated balloons. The recommendation to use both DES and drug-coated balloons has therefore long been included in cardiology guidelines [16, 17].

DES have been used to treat symptomatic ICAD in the pre-SAMMPRIS, pre-VISSIT era, and preceding studies showed promising results [11, 18–25]. However, SAMMPRIS and VISSIT caused a remarkable and sustained decline in industrialized research of intracranial stenting materials and procedures. It is only recently slowly being restarted, after the re-evaluation of the treatment recommendations drawn from SAMMPRIS and VISSIT has been called for on the basis of the medical progress made in the meantime [2].

In support of the mentioned trends, we therefore performed a direct comparison of the short- and mid-term outcome of patients electively treated for symptomatic ICAD in the anterior circulation using conventional stents and DES.

We specifically chose the Coroflex® ISAR NEO stent for closer examination. In the few pre-SAMMPRIS/-VISSIT studies, the rigidity and poor controllability of primary cardiac stents was described [20]. The Coroflex® ISAR NEO is supposed to be a further development in terms of flexibility and controllability compared to its predecessors.

The second objective was therefore to find out whether the Coroflex® stent can meet its claims in the treatment of ICAD and early ISRS in a larger number of patients than previously published.

## Methods

### Patient population

We investigated the outcome of all patients who underwent elective treatment of intracranial arterial stenosis by stent angioplasty in our department between May 2014 and June 2022. Age, gender, relevant pre-existing conditions such as hyperlipidemia and diabetes, tobacco consumption and previous stroke were recorded. The pre-operative modified rankin scale (mRS) score was recorded.

Inclusion criteria for this study were.

- Age over 18 years.
- Written consent to the procedure.
- Symptomatic, drug-refractory intracranial arterial stenosis in the anterior circulation (intracranial internal carotid artery ICA, middle cerebral artery MCA): i. e. symptoms despite >10d of previous best medical treatment (BMT)
- High risk of stroke due to the stenosis in a planned otherwise vascular treatment (e. g. patients experiencing ICAD symptoms, most likely due to hypotensive situations caused by a heart disease requiring urgent invasive treatment)

BMT consisted of antiplatelet therapy monitored through point-of-care response tests, lipid-lowering therapy with statins, and antihypertensive medication.

The cut-off of >  10 d BMT was chosen to assure the BMT to be in full effect and to not treat symptomatic patients whose effective level of the medication had not yet been reached.

### Drug regimen

The drug regimen consisted of acetylsalicylic acid (ASA) at 100 mg/d and ticagrelor at 180 mg/d, or prasugrel at 10 mg/d, or clopidogrel at 75 mg/d and was administered at least 5 days before the procedure. Sufficiency of platelet inhibition was assessed in advance with a Multiplate Analyzer (MP; Roche Diagnostics, Mannheim, Germany) and VerifyNow (VN; Accriva, San Diego, CA, USA) on the day of the treatment. In the event of inadequate platelet inhibition, the relevant medication dosage was adjusted accordingly, and the treatment was postponed. In the event of inadequate response or intolerance to any of the agents, the drug was changed to another agent described above. Dual antiplatelet therapy (DAPT) was continued for at least 3 months, and then switched to lifelong ASA at 100 mg/d.

### Endovascular treatment

The degree of stenosis before and after angioplasty was determined using the WASID method [26], in which the quotient of the vessel diameter in the stenosed segment and the proximal physiological diameter was calculated. The result was subtracted from 1 to obtain a percentage value.

All procedures were performed under general anesthesia. Access was obtained via the femoral or radial artery. All patients received an intravenous bolus injection of 3000 IU non-fractionated heparin sodium directly after the sheath insertion.

A guide catheter (e.g. 6 F Envoy MPC, Cerenovus) was placed in the proximal internal carotid artery ipsilateral to the stenosis. Under Roadmap guidance, the stenosis was passed with a 0.014-inch micro-wire (e. g., Synchro2, Stryker; pORTAL, WallabyPhenox) and the distal vessel segment was probed. If necessary, the stenosis was carefully pre-dilated using a balloon catheter (e. g., pITA, WallabyPhenox). Then, a 0.021 inch inner diameter microcatheter (e.g., Phenom21, Medtronic; Rebar-18, Medtronic; or Trevo pro 18, Stryker) was advanced distal to the stenosis. The stent system was then deployed into the stenotic segment via the catheter by either retraction of the microcatheter alone (for self-expanding systems) or by direct probing of the stenotic segment using a micro-wire and balloon stent system and inflation of the balloon on which it is mounted (for Coroflex® ISAR NEO) – the latter step without the usage of a microcatheter. All stents were willingly undersized.

The procedure was considered successful if a stenosis reduction of more than 40% using the above-mentioned method of measuring could be achieved.

In stenting of the proximal carotid artery, embolic protection devices can be beneficial in reducing DWI-lesions in post-procedural MRI [27]. However, to the authors’ knowledge, so far there is no device available that would offer a comparable degree of flexibility and safety for intracranial stenting.

Theoretically, to achieve a degree of embolic protection in intracranial stenting with existing devices, a balloon guide catheter could be inflated in the cervical ICA for flow-arrest prior to stenting. After the stent-assisted angioplasty and still under flow-arrest, the stented vessel could be passed through with an aspiration catheter under constant proximal aspiration for reduction of any clot and debris before eventually restoring antegrade flow. However, we decided against this manoeuver due to its higher complexity and risk of complications, e.g. stent displacement/disfigurement through the aspiration catheter etc.

The decision as to whether a Coroflex® stent or a self-expandable stent should be implanted was based on the individual vessel anatomy and the individual degree of vessel degeneration. In that sense, BMS was chosen for the supraophthalmic ICA segment and proximal to middle MCA if the proximal vessel anatomy seemed too tortuous for the comparably stiffer Coroflex® stent. The petrous and cavernosal ICA segments however did not have such a criteria of decision. Ultimately, the decision was to the discretion of the head physician in our department with over 30 active work years/experience in neuroendovascular intervention.

### Follow up

Every patient underwent pre-operative examination and mRS scoring through a board certified neurologist. Peri-operative complications and any deterioration within 10 days after intervention (in-hospital phase) were followed-up and documented. Before discharge from the hospital, all patients received neurological examination and underwent a brain magnetic resonance imaging (MRI) scan, including T2-fluid-attenuated inversion recovery (FLAIR) and diffusion-weighted imaging (DWI) sequences.

Subsequently, digital subtraction angiography (DSA) of the treated vessel was performed 90 days (3MO) after treatment and all patients were again examined and cored by a board certified neurologist. The degree of stenosis was determined on DSA using the WASID method described above.

All interim ischemic or hemorrhagic events were documented, the mRS score was recorded and the effectiveness of DAPT was measured. If the DAPT effect was too strong or too weak, the dose was adjusted accordingly.

### Statistical analysis

Continuous data were described as the mean, median, minimum, and maximum. Numbers and percentages were used to describe categorical data. The quartiles of the group were created and the interquartile ranges were calculated as a measure of statistical dispersion. Fisher’s exact test was fit on the data for null-hypothesis significance testing; it is described as p-value with a level of significance if < 0.05.

SPSS26 (IBM, Armonk, united states of America) was used for the statistical analysis.

## Results

**Fig. 1:**
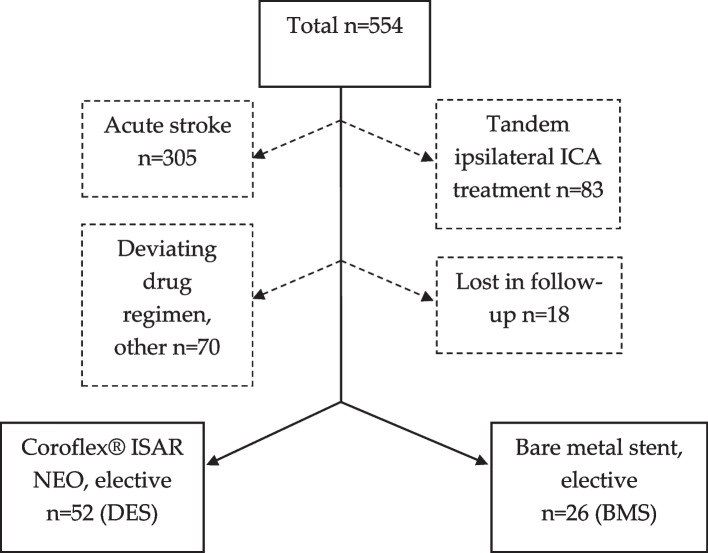
Treatment figures of intracranial, intradural ICAD treatment in the anterior circulation via stent-PTA 2014–2022

In the mentioned period, 554 intracranial ICAD treatments through stent- angioplasty were performed at our department. Of these, seventy-eight patients (n male: female = 57: 21) were included in the study, 52 patients received treatment with the Coroflex ISAR NEO stent, 26 patients were treated with non-coated, self-expanding stents (23 × Enterprise, Codman Neurovascular, USA; 2 × Solitaire AB, Medtronic, USA; 1 × Neuroform Atlas, Stryker Neurovascular, USA); *s.* (Fig. 1).

Stent diameters and length ranged from 2 mm/9 mm to 4 mm/16 mm for the Coroflex ISAR NEO DES.

For the Codman Neurovascular Enterprise BMS, used stent diameters and lengths ranged from 4 mm/14 mm to 4,5 mm/30 mm. The applied Solitaire AB BMS had a diameter and length of 3 mm/30 mm and 5 mm/20 mm, the diameter and length of the Neuroform Atlas BMS was 4 mm/15 mm.

Applied balloon catheters were the WallabyPhenox pITA wit length and diameter ranging from 1,5 mm/10 mm to 4 mm/40 mm, the Terumo Hiryu balloon catheter ranging from 2,25 mm/20 mm to 3 mm/20 mm in diameter and length, and the Terumo Ryurei balloon catheter, ranging from 1 mm/5 mm to 3,5 mm/40 mm in diameter and length.

**Fig. 2:**
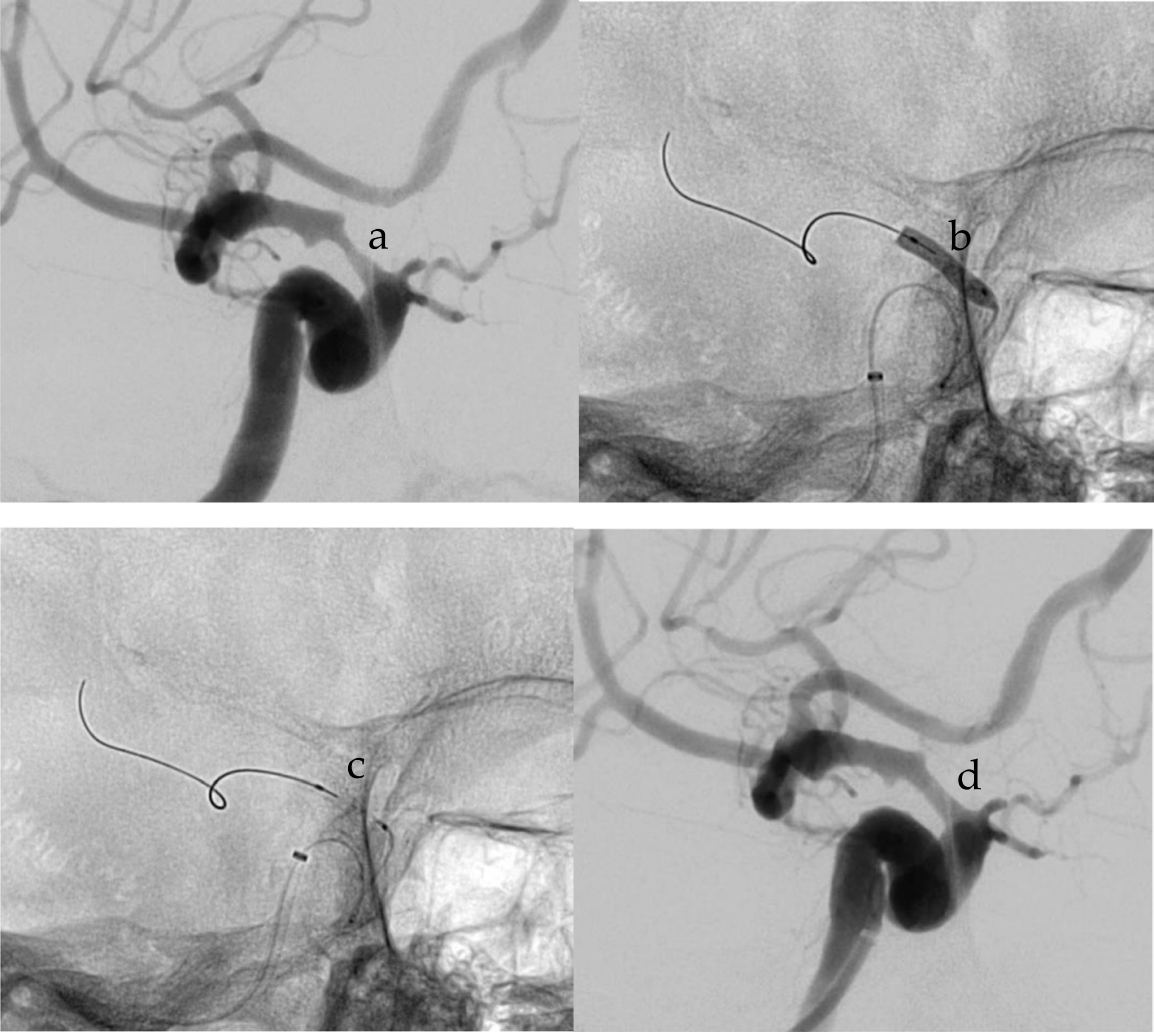
Stenosis of the supraophthalmic internal carotid artery before treatment (**a**), under balloon-dilation (**b**), with stent implanted (**c**) and angiogram following implantation (**d**)

Mean age was 69,2 years (47-87y, IQR 12), the mean age in the subgroups was 68,9 years in the DES-group (47-87y, IQR 11,5) and 69,7 years in the BMS-group (48-85y, IQR 15,75) (Fig. 2).

Arterial hypertension was the main comorbidity in both groups with 38% in DES and 42% in BMS, followed by coronary heart disease with 31% in DES and 30% in BMS. Diabetes mellitus was present in 25% of DES patients vs. 38% in the BMS group. Ten percent of patients were current tobacco smokers in DES vs. 17% in BMS (Table 1).

**Table 1:** Comorbidities of the study population, in order of frequency in the DES group. *body mass index

Comorbidity	DES group (% of population)	BMS group (% of population)	p-value
Arterial hypertension	38,4	42,3	0.113
Coronary heart disease	30,8	30,8	0.983
Diabetes mellitus	25	38,5	0.165
Hyperlipidemia	21,2	30,8	0.317
Current tobacco smoker	10	16,6	0.192
Obesity (BMI* < 30)	3,8	7,7	0.226

Sixty-three patients suffered a stroke or TIA within 30 days prior to the procedure under BMT for at least 10 days. Twelve patients were treated due to impending ipsi- or contralateral cervical carotid intervention with contralateral chronic occlusion (n = 5), insufficient relief through external-intracranial bypass at chronic contralateral medial cerebral artery stenosis/occlusion (n = 2) and at the express request of the patient to avoid the psychological stress of knowing the diagnosis of ICAD (n = 1). The mRS score prior to intervention was 0–2 in 88% of DES patients (n = 46) vs. 73% in BMS, 3–4 in 12% (n = 6) of DES-patients vs. 23% (n = 6) in BMS. One patient in BMS was scored mRS 5 prior to intervention.

The location of stenosis was predominately the petrous segment of the ICA (49% in DES vs. 50% in BMS), followed by the ICAs’ cavernous segment with 21% in DES vs. 8% in BMS, and the supraophthalmic ICA segment and proximal to middle MCA with 29% in DES and 42% in BMS. Mean degree of stenosis before treatment was 78,7% (68–97%, IQR 11,75) in total, in subgroups 79,3% in DES vs. 77,1% in BMS (IQR DES 10 vs. 15,3 in BMS).

In the DES-group, 56% of patients required pre-angioplasty via percutaneous transluminal balloon-catheter. In the BMS-group, due to the fact that the deployed stents were all non-balloon-mounted, self-expandable, predilation was performed in 92% of cases, post-dilation in 8%. Regarding the stenosis reduction, the intervention was successful in 100% of patients. In the DES-group, two patients suffered from clinically apparent, yet minor periprocedural stroke raising the post-treatment (≤ 10 days after stenting) mRS score temporarily by 1 point each (mRS 0—> 1 at discharge—> 0 at 3mo follow-up). In the BMS-group, four minor and one major intraprocedural/postprocedural complication occurred:

- One patient suffered from major intraprocedural stroke, raising his mRS permanently from mRS 1 to mRS 3
- Three patients suffered from minor stroke, temporarily raising their mRS from mRS 0 to mRS 1
- In one patient, an intraprocedural stent-dislocation occurred, making it necessary to implant another stent in the stenotic section of the vessel. This incidence was rated as a complication, but did not affect the patients mRS

In all other patients, the mRS score pre- and post-treatment was equal or better. However, on MRI before discharge, 50% (n = 26) of patients in DES and 62% (n = 16) of patients in BMS showed new treatment-related, clinically inapparent ischemic lesions on DWI.

All patients could be followed up with DSA after 3 months. The mean time of follow-up was 102 days in the DES group (82–140 d) and a mean of 109 days (71–131 d) in the BMS-group. Recurrent stroke in the territory of the stented vessel had occurred in two patients (3,8%) in the DES stent group vs. three patients (11,5%) in the BMS-group. This resulted in a mRS score rise of 2 to 4 in one patient in the DES group vs. a rise of one point each in the BMS-group. However, recurrent stroke was associated with ISRS in only one case of a BMS –patient.

ISRS (> 60% following WASID) occurred in 2 patients (3,8%) in the DES group vs. 9 patients (34,6%) in the BMS-group.

Eight patients were treated for ISRS via drug-coated balloon angioplasty; one patient was treated with balloon-assisted re-stenting. One patient underwent endovascular ISRS treatment outside our hospital. The main results of the two groups are compared in Table 2.

**Table 2:** Main results of the DES and BMS group in comparison

	DES	BMS	p-value
Mean Stenosis grade (WASID)	79,3%	77,1%	0.527
Balloon-angioplasty preceding stenting	56%	92%	< 0.001
Periprocedural complication	3,8%	19,2%	0.015
Recurrent stroke within 90d	3,8%	11,5%	0.093
ISRS	3,8%	34,6%	< 0.001

## Discussion

The aim of this study is to determine the treatment options for ICAD using the Coroflex® ISAR NEO and its performance in terms of early ISRS compared with uncoated, conventional stents. Apart from recurrent stroke, ISRS was a main reason for the shift from endovascular treatment to BMT as first line ICAD treatment after SAMMPRIS and VISSIT. Only BMS were used in both endovascular arms of these studies. We therefore focuses on approaches to challenge ISRS also in the neurovascular field by comparing the endovascular arms of VISSIT and SAMMPRIS with this study.

A comparison of the patient data and comorbidities of the DES and BMS groups reveals a relatively similar patient population. Even in the group of diabetics with the greatest distance between BMS and DES, there is no statistical significance.

However, looking at the rates of periprocedural ischemic events, the rate in the DES group is significantly lower. Both groups were always accompanied by balloon dilatation, in the DES group due to the fact that the Coroflex® ISAR NEO stent is balloon-supported, and in the BMS stent group due to the predilatation required in almost all cases.

Balloon angioplasty can lead to the so-called snowplow phenomenon, a process in which plaque material is pushed through the balloon into the outlets of small perforators, leading to microinfarcts that are relevant in the aggregate [28]. This type of pathology with the underlying phenomenon was more pronounced in VISSIT in comparison between SAMMPRIS and the VISSIT study, in which only balloon-supported stents were used [14]. The WEAVE study and other studies were able to show that submaximal dilatation of the corresponding vessel segment led to a lower number of periprocedural infarctions during ICAD treatment using balloon angioplasty and stenting [29]. Until 2019, in the lack of the study data, and then based on the results of the WEAVE trial, we selected undersized stents in both the BMS and DES groups from the outset.

Ultimately, the predilation rate of 92% is higher in the BMS group than in the DES group, which could explain the difference in periprocedural ischemia due to the snowplow effect. However, since the DES are also released by balloon dilatation, the proportion of this potentially complication-prone maneuver is higher in the DES group. The fact that this is not reflected in a higher ischemia rate may be attributed to the inequality of the study groups and a slightly higher amount of BMS deployment in the more complex region of distal ICA and proximal to middle MCA-segment. However, as the statistical analysis shows a clear significance, the authors also suspect a positive systemic effect due to the lower complexity of the maneuver with a balloon-mounted stent that does not require a catheter exchange step after predilation.

In SAMMPRIS, beyond 30 days, 10% of patients in the conservative as well as in the endovascular arm suffered from recurrent stroke and/or death [30]. This rate was exceeded in the VISSIT trial with 24,1% [14] within 30 days, which ultimately even led to the premature end of the study. The rate of recurrent strokes within the triple time period in the BMS group of the present study rather reproduces the SAMMPRIS results, whereas it is clearly lower, yet not statistically significantly lower in the DES group. This lower rate could be related to the likewise and statistically significantly lower ISRS rate in the DES group. Although the stroke events in our study group were not significantly frequently associated with ISRS, it is possible that a reduction in endothelial irritation due to the drug-induced stent coating already leads to protection of the perforators of the covered segment or reduced endothelial irritation with a corresponding reduction in the release of inflammatory factors.

In cardiology, the ISAR-STEREO trial started the assumption that the thickness of the stent struts has a relevant effect on the success of treatment [31]. This led to the development from thick-strut- to thin- to ultra-thin strut stents in cardiology, related to the hypothesis that thicker stent struts lead to delayed endothelialization [32] and thus maintain a risk of thrombosis for longer [33, 34] than a stent with thinner struts and correspondingly faster incorporation into the vessel wall might do.

This hypothesis refers to BMS, but ultimately the stents currently approved for ICAD use may already benefit from this cardiological experience, as the stent strut thickness qualifies all BMS stents approved for ICAD as ultra-thin stents (= average strut thickness < 70 µm). At 55—65 μm, the strut thickness of the Coroflex® ISAR NEO system lies between that of the other BMS systems used, although not below other market-ready DES [35].

In cardiology, however, the combination of ultra-thin stents with drug coating is also regarded as critical. A conspicuously high rate of fractures and late in-stent thrombosis of DES in cardiology settings is a cause for concern [36]. A sequence of delayed endothelialization due to the high effectiveness of sirolimus in inhibiting endothelial proliferation is seen as a possible cause. This could lead to a higher susceptibility to stent fractures due to the lack of a stabilizing effect of the vascular wall insertion and/or promote platelet aggregation in the smallest areas between the stent and the vascular wall [37]. Both could ultimately lead to the conspicuous rate of late adverse cardiac events or ISR or in-stent thrombosis [38–40].

However, the follow-up times in the current literature for ICAD treatment using DES and also their case numbers are in some cases far below the studies [41], on which the cardiological findings are based. The structure of the intradural vessels also differs due to the absence of the membrana elastica externa and the reduced thickness of the muscular tunica media. In addition, intradural arteries are not subject to the dynamics of a myocardial artery. Based on these points, it is not yet possible to say whether the experience gained in cardiology can also be translated to neuroradiology.

Regarding preceding studies on the use of DES in ICAD treatment, data is sparse. However, three recent randomized controlled trials provide a good insight into the trend in the outcome of ICAD treatment using DES and show comparability in individual areas with this study: Jia et al. [11] reported the results of a DES vs. BMS comparison in 132 vs. 131 patients in a multicenter study in 2022. The inclusion criteria, patients’ clinical data and eluted drug are comparable with data in this study. However, Jia et al. also included treatments of the posterior circulation and a different kind of DES with was used. He et al. [42]compared the results of DES vs. BMS in the vertebral artery in 20 vs. 20 patients. Again, inclusion criteria, and patients’ clinical data are vastly comparable with our data, sirolimus is the eluting drug of the stent. Yet, the posterior circulation is a major difference to this study. Si et al. [43]report the results of 92 DES vs. 96 BMS in the treatment of ICAD in both the posterior and anterior circulation. Again, patients’ clinical data, inclusion criteria and Sirolimus as eluted drug are comparable. However, inclusion of treatments in the posterior circulation is a major difference. Table 3 displays the main results:

**Table 3:** Main results of preceding studies in comparison to data in this study on DES vs. BMS

Study	ISRS	Recurrent stroke	n DES
He et al. 2019 [42]	5% at 6 months	0% in 12 months	20
Si et al. 2022 [43]	14,5% at 3 months	2,2% in 12 months	92
Jia et al. 2022 [11]	9,5% at 12 months	0,8% in 12 months	132
Data in this study	3,8% at 3 months	3,8% in 90d	52

Following the publication of the SAMMPRIS and VISSIT results, research into ICAD stent treatment slowed down or even ceased. In this context, there are still no guidelines or declarations of consent that would recommend or favor a particular type of stent for ICAD treatment. However, based on advanced knowledge of the pathology behind ISRS in cardiology, the authors agree with those who advocate the use of drug-coated implants for the treatment of ICAD as well.

### Strengths and weaknesses

The weaknesses of this study clearly lie in the numerical imbalance of the two groups DES vs. BMS. In addition, three different models were examined in the BMS group compared to only one model in the DES group. Combined with a slight overrepresentation of BMS in intradural vascular segments, this probably results in a bias regarding the strikingly high rate of periprocedural complications in the BMS group. However, the rate of ISRS for the BMS group is similar to that described in the literature, and the rate of recurrent stroke in the BMS group also appears to be in line with the results of other studies—which ultimately suggests that the bias due to numerical imbalance may be moderate.

The follow-up time in this study does not allow the assessment of a therapeutic effect of DES vs. BMS. However, SAMMPRIS and VISSIT did not assess the therapeutic effect either, and the recommendation against endovascular ICAD treatment as first line therapy resulted from the higher complication rate in the early phase following treatment.

In addition, there could be a selection bias in the present study, as the decision to implant BMS or DES was not based on chance, but on the decision of the most senior and experienced neurointerventionalist in the department. This point is part of the weakness of this study through the retrospective design and single center setting.

The strengths of the study are however the—to the authors' knowledge—unprecedentedly high number of cases in the DES-BMS comparison for a single-center study with the correspondingly homogeneous conditions. The time interval of 90 days is also rarely found for DES-BMS-comparative studies within this setting. Finally, the fact that only one dedicated DES model was examined may also have a positive impact on the significance of the study results due to the homogeneity it creates.

## Conclusion

In the population studied, the DES was shown to be comparable or clearly advantageous compared to the BMS in terms of ISR and periprocedural complication rates. Based on these results and in the light of further data on ICAD treatment by DES from the recent past, the authors therefore clearly advocate a re-evaluation of endovascular ICAD treatment as a first-line therapy.

However, long-term results of ICAD treatment using DES are urgently needed to avoid transferring the deficits of DES described in cardiology to the neurovascular setting.

## Data Availability

All published data was collected by the group of authors; no data subject to third-party copyright was published. The datasets used and analyzed during the current study are available from the corresponding author on reasonable request.

## Abbreviations

BMI: Body mass index
BMS: Bare-metal stent
BMT: Best medical treatment
DAPT: Dual antiplatelet therapy
DES: Drug-eluting stent
DSA: Digital subtraction angiography
DWI: Diffusion-weighted imaging
FLAIR: Fluid-attenuated inversion recovery
ICA: Internal carotid artery
ICAD: Intracranial atherosclerotic disease
IQR: Interquartile range
ISRS: In-stent restenosis
MCA: Middle cerebral artery
MRI: Magnetic resonance imaging
